# Neutralizing nanobodies against porcine epidemic diarrhea virus: discovery and characterization

**DOI:** 10.1186/s12917-026-05628-z

**Published:** 2026-07-23

**Authors:** Mengya Fan, Xuran Wang, Yangyang Yan, Lili Wang, Jiayi Wei, Guohu Chen, Yufei Wang, Junlan Zan, Lianping Xu, Fengsai Li, Shijie Yan, Shanshan Yang, Ping Rui, Zengjun Ma, Wei Liu, Tao Song

**Affiliations:** 1 Hebei Key Laboratory of Preventive Veterinary Medicine, College of Animal Science and Technology, Hebei Normal University of Science and Technology, Qinhuangdao, 066600 China; 2 Key Laboratory of Prevention and Control Agents for Animal Bacteriosis (Ministry of Agriculture and Rural Affairs), Hubei Provincial Key Laboratory of Animal Pathogenic Microbiology, Institute of Animal Husbandry and Veterinary, Hubei Academy of Agricultural Sciences, Wuhan, 430070 China; 3 Bayingol Mongolian Autonomous Animal Disease Control and Diagnosis Center, Kurle, 841000 China

**Keywords:** Porcine epidemic diarrhea virus, Nanobody, Phage display, Neutralizing antibody, Spike protein

## Abstract

Porcine epidemic diarrhea virus (PEDV) causes severe mortality in piglets, inflicting significant economic losses globally. Nanobodies (Nbs) offer distinct advantages over conventional antibodies for disease intervention. This study aimed to develop neutralizing Nbs against the PEDV spike (S) protein. We constructed a eukaryotic plasmid encoding the S protein of the CH-HB2-2018 strain and expressed it in HEK-293 F cells. Following alpaca immunization with the purified S protein, a phage display library was constructed. Biopanning yielded 70 unique Nbs based on CDR diversity, with 67 confirmed as specific binders via ELISA and Western blot. Further characterization using immunofluorescence and flow cytometry identified 9 specific candidates. Microneutralization assays revealed that these candidates showed varying degrees of inhibition against PEDV replication in Vero cells. Notably, nanobody 3Nb17 exhibited the highest binding affinity and neutralizing titer. These findings highlight 3Nb17 as a promising candidate for developing novel PEDV diagnostic tools and therapeutics.

**Supplementary Information:**

The online version contains supplementary material available at 10.1186/s12917-026-05628-z.

## Introduction

Porcine Epidemic Diarrhea Virus (PEDV), a highly contagious *Alphacoronavirus* within the family Coronaviridae, has inflicted substantial economic losses on the global swine industry over the past decade. The virus causes acute enteric disease characterized by severe dehydration and high mortality, particularly in newborn piglets [1, 2]. Despite the widespread application of vaccines, PEDV outbreaks remain frequent and difficult to control. This failure is largely attributed to the rapid genetic evolution of the virus, especially in the spike (S) protein, which compromises the cross-protection efficacy of traditional immunoprophylaxis [3]. Consequently, the development of novel, broad-spectrum, and stable antiviral agents is urgently needed to complement existing control strategies.

The homotrimeric S glycoprotein is the primary determinant of viral tropism and pathogenicity. It comprises two subunits: S1, responsible for binding to the aminopeptidase N (APN/CD13) receptor on porcine intestinal epithelial cells, and S2, which mediates membrane fusion [4, 5]. Unlike antibodies against the nucleocapsid (N) protein, which are abundant but non-neutralizing and may even induce antibody-dependent enhancement (ADE) of infection [6, 7], the S protein-specifically its S1 subunit-contains critical neutralizing epitopes [8, 9]. However, the S protein is heavily glycosylated and undergoes post-translational modifications such as palmitoylation, which shield key epitopes and stabilize the viral structure against host immune surveillance [10, 11]. Therefore, targeting the S protein requires high-affinity binders capable of navigating these structural defenses.

Nanobodies (Nbs), or single-domain antibodies (VHHs) derived from camelid heavy-chain-only antibodies, offer a superior alternative to conventional monoclonal antibodies (mAbs) for targeting complex viral glycoproteins [12]. While conventional mAbs often struggle with tissue penetration and high production costs [13], nanobodies possess unique structural features that are advantageous for biotechnological applications. Their small size (~ 15 kDa) and long CDR3 loops allow them to access cryptic, conserved epitopes often hidden within clefts or shielded by glycans on the viral surface [14]. Furthermore, nanobodies exhibit exceptional stability under harsh thermal and chemical conditions, making them ideal for development as orally administered or aerosolized therapeutics for livestock [15]. Their modular nature also facilitates the engineering of multivalent constructs to enhance binding avidity and prevent viral escape [16].

In this study, we aimed to harness these advantages by developing specific neutralizing nanobodies against PEDV. We constructed a high-diversity phage display library from an alpaca immunized with recombinant PEDV S protein. Through rigorous biopanning and functional characterization, we identified a novel nanobody, 3Nb17, which exhibits potent neutralizing activity and high binding affinity. This candidate provides a promising foundation for the development of next-generation diagnostic reagents and therapeutic interventions against PEDV infection.

## Materials and methods

### Cells and reagents

HEK-293 F (ATCC CRL-3249), HEK-293T (ATCC CRL-3216), and Vero (ATCC CCL-81) cells were maintained in our laboratory. HEK-293 F cells were cultured in suspension in SMM 293-TII medium (Sino Biological, Beijing, China). HEK-293T and Vero cells were maintained as adherent cultures in DMEM (Gibco) supplemented with 10% fetal bovine serum (FBS, Gibco) and 1% penicillin-streptomycin. All cells were incubated at 37℃ in a 5% CO_2_ atmosphere. TG1 electrocompetent cells were purchased from Lucigen (USA). The endotoxin-free plasmid extraction kit was obtained from TIANGEN (Beijing, China). Sinofectin transfection reagent were purchased from Sino Biological (Beijing, China). The anti-His mAb (mouse monoclonal, clone 1B7G5, catalog No. 66005-1-Ig, Proteintech, Chicago, IL, USA) was used at a dilution of 1:5000 for Western blot. The HRP-conjugated anti-6×His tag antibody (clone EPR20547, catalog No. ab1187, Abcam, Cambridge, UK) was used at a dilution of 1:5,000 for ELISA detection of His-tagged proteins in the periplasmic extraction ELISA (PE-ELISA). The FITC-conjugated goat anti-mouse IgG secondary antibody (catalog No. AS001, Abclonal, Wuhan, China) was used at a dilution of 1:500 for indirect immunofluorescence assay (IFA). The FITC-conjugated anti-His tag antibody (catalog No. ab1206, Abcam, Cambridge, UK) and the APC-conjugated anti-mouse Fc antibody (catalog No. AB99901, Abcam, Cambridge, UK) was used at a dilution of 1:100 for flow cytometry analysis. The PEDV-positive pig serum used as a positive control was generously provided by Professor Bin Li (Institute of Veterinary Medicine, Jiangsu Academy of Agricultural Sciences), which was obtained from pigs immunized with a PEDV-spike-protein-expressing mRNA vaccine as previously described [17].

### Recombinant antigen preparation

A codon-optimized gene encoding the PEDV S protein (strain CH-HB2-2018, GenBank: MK606369) containing an N-terminal gp67 signal peptide, a T4 foldon trimerization tag, and a C-terminal 6×His tag was cloned into the pCAGGS vector. The recombinant plasmid pCAGGS-PEDV-S was transfected into HEK-293 F cells at a density of 2–3 × 10^6^ cells/mL. For 200 mL of culture, 400 µg of plasmid and 400 µL of Sinofectin transfection reagent were each diluted in 10 mL serum-free medium, mixed, incubated for 15 min at RT, and added to the cells. Culture supernatants were harvested 5 days post-transfection. The S protein was purified using a HisTrap Excel affinity column followed by size-exclusion chromatography (SEC) on a HiLoad Superdex 200 column (Cytiva). Purified proteins were denatured at 95℃ for 5 min in SDS sample buffer containing 50 mM DTT (reducing condition), separated by 10% SDS-PAGE, and visualized by Coomassie blue staining and Western blot using an anti-His mAb (Proteintech).

### Alpaca immunization and library construction

A healthy adult alpaca was provided by the Qinhuangdao Wildlife Park (Qinhuangdao, China). Formal authorization for the use and immunization of the alpaca was explicitly granted by the park management. The alpaca was immunized subcutaneously four times at bi-weekly intervals with 400 µg of purified S protein mixed with the MF59 adjuvant [18]. One week after the 4th boost, PBMCs were isolated for total RNA extraction. The VHH gene repertoire was amplified via nested PCR (using primers CALL001/CALL002 for the first round, and VHH-F(1–4)/VHH-R(1–3) for the second round; all sequences are listed in Table 1) and cloned into the pComb3XSS phagemid vector. The ligation products were electroporated into *E. coli* TG1 cells to generate the phage display library. Library size was estimated by colony counting, and insertion efficiency was verified by colony PCR using primers S-F and S-R (Table 1). Since the experimental procedures were strictly limited to routine immunizations and a single blood collection, no animals were euthanized or sacrificed in this study. Following the final blood draw, the alpaca remained healthy under the continuous care of the park management.

**Table 1:** Primers used for VHH library construction and colony PCR screening

Primer name	Primer sequence(5’~3’)
CALL001	GTCCTGGCTGCTCTTCTACAAGG
CALL002	GGTACGTGCTGTTGAACTGTTCC
VHH-F1	AGAATGGCCCAGGCGGCCSAGGTGCAGMTGCAGGASTCGGGCCCA
VHH-F2	AGAATGGCCCAGGCGGCCCAGGTCCAGYTGGTGCARCCAGGGGCT
VHH-F3	AGAATGGCCCAGGCGGCCSAGGTGCAGRTGGTGGASTCTGGGGGA
VHH-F4	AGAATGGCCCAGGCGGCCCAGMTGCAGCTCGTGGADTCTGGGGGA
VHH-R1	CCTGGCCGGCCTGGCCTGAGGAGACMGTGACCRGGGT
VHH-R2	CCTGGCCGGCCTGGCCTSAGGACACGGTGCCCRGGTG
VHH-R3	CCTGGCCGGCCTGGCCTSAGGAGACGGTGACCTGGGT
S-F	TATGTTGTGTGGAATTGTGAGCGG
S-R	GTTTGCCATCTTTTCATAATCAAAATCAC

### Phage display biopanning

Specific nanobodies were enriched through three rounds of biopanning against immobilized S protein. To select for high-affinity binders, the antigen coating concentration was sequentially reduced (2, 1, and 0.5 µg/mL). Phages were eluted with 100 mM glycine-HCl (pH 2.2) and neutralized with 1 M Tris-HCl (pH 7.4). Enrichment was monitored by calculating the output-to-input phage ratio.

### Screening and identification of nanobodies

Individual colonies from the third round were grown in 96-well plates, and periplasmic extracts were prepared for PE-ELISA screening [19]. Positive clones (signal-to-noise ratio > 2.1) were sequenced. Unique nanobodies were identified based on CDR3 diversity alignment using MEGA 11 software. Selected nanobodies were expressed in *E. coli* WK6 cells and purified via Ni-NTA affinity chromatography for further characterization.

### IFA and flow cytometry

For indirect immunofluorescence assay (IFA), the VHH gene in pCAGGS-VHH-mFc (mouse IgG Fc tag) was transfected into HEK-293T cells. Cells were seeded to 70–80% confluence one day prior. Per well (12-well plate), 1 µg DNA in 50 µL serum-free medium was mixed with 2 µL Sinofectin transfection reagent, incubated for 15 min at RT, and added to cells. Supernatant was harvested 48 h post-transfection for IFA. The supernatants containing VHH-Fc fusions were incubated with PEDV-infected Vero cells (fixed and permeabilized), followed by staining with FITC-conjugated goat anti-mouse IgG. Serum from PEDV-vaccinated pigs was used as the primary antibody for the positive control, while serum from healthy, unvaccinated pigs served as the negative control.

For flow cytometry analysis, HEK293T cells were transfected with the pCAGGS-PEDV-S-his plasmid expressing the full-length PEDV S protein. Post-transfection, the cells were harvested and incubated with the purified nanobodies at 4℃ for 30 min. To detect the specific binding interactions, the cells were subsequently washed and incubated with an anti-His FITC-conjugated antibody and an APC-conjugated anti-mFc antibody. Serum from PEDV-vaccinated pigs was used as the positive control, while an irrelevant nanobody as the negative control. The samples were then analyzed using a FACSAria III flow cytometer (BD Biosciences).

### Virus neutralization assay

The neutralizing activity of nanobodies was determined by a microneutralization assay on Vero cells. The virus titer (TCID_50_) was determined by the endpoint dilution method [20] and calculated using the Reed-Muench method [21]. Serial dilutions of nanobodies were mixed with 200 TCID_50_ of PEDV CH-HB2-2018 and incubated for 1 h at 37℃. The mixture was added to Vero cell monolayers. Cytopathic effects (CPE) were observed at 48 h post-infection. The 50% inhibitory concentration (IC_50_) was calculated using GraphPad Prism 9.0.

### Surface Plasmon Resonance (SPR)

Affinity kinetics were measured using a Biacore T200 instrument (Cytiva). Purified PEDV S protein was immobilized on a CM5 sensor chip via amine coupling. Nanobodies were injected at various concentrations (ranging from 1.56 to 50 nM). Kinetic parameters (*K*_on_, *K*_off_, and *K*_D_) were derived by fitting the sensorgrams to a 1:1 Langmuir binding model [22].

## Results

### Preparation of recombinant PEDV S protein

**Fig. 1:**
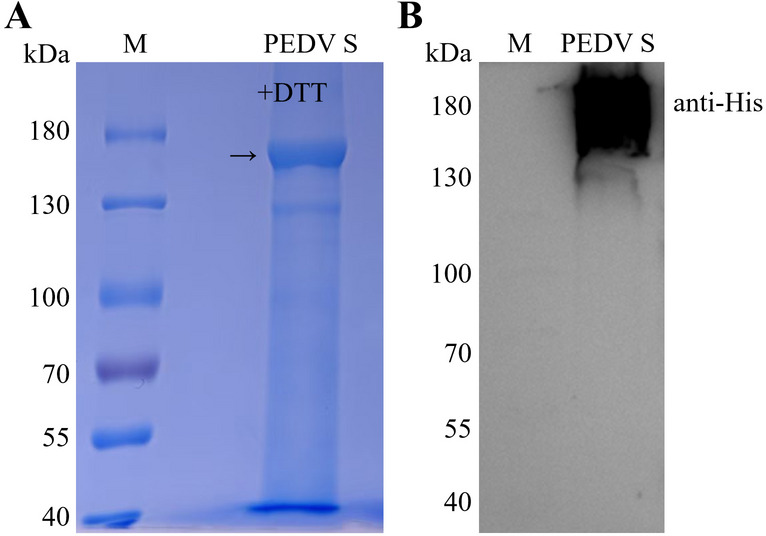
Preparation and verification of the recombinant PEDV S protein. **A**: SDS-PAGE analysis of the purified S protein expressed in HEK-293 F cells. Reducing SDS-PAGE analysis of purified PEDV S protein. The major band at ~ 180 kDa corresponds to the monomeric form. M: Protein molecular weight marker. **B**: Western blot analysis of the purified S protein using an anti-His tag monoclonal antibody

The recombinant S protein was successfully expressed in HEK-293 F cells and purified to high homogeneity. SDS-PAGE analysis under reducing conditions revealed a major band migrating at approximately 180 kDa, consistent with the expected molecular weight of the monomeric glycoprotein (Fig. 1A). Western blot analysis using an anti-His antibody confirmed the specificity of the purified protein (Fig. 1B). The high purity of the antigen laid a solid foundation for subsequent immunization and screening.

### Construction of the immune VHH library

**Fig. 2:**
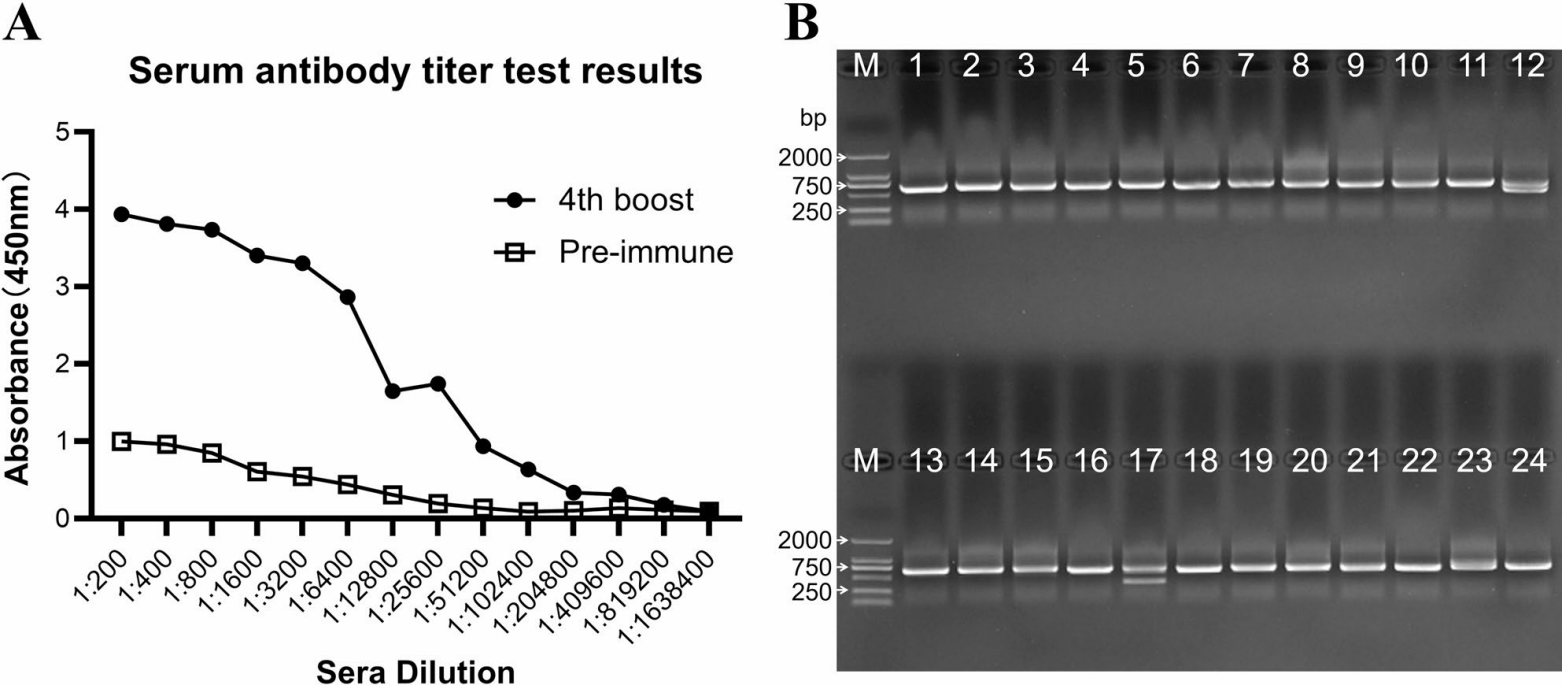
Construction and quality assessment of the immune VHH phage display library. **A**: Serum antibody titer of the immunized alpaca against PEDV S protein determined by ELISA. **B**: Colony PCR verification of VHH gene insertion efficiency. M: DL2000 DNA molecular weight marker; 1–24: Randomly selected clones of the phage display library

Following four immunizations, the alpaca elicited a robust serum antibody response against the PEDV S protein, with titers significantly exceeding pre-immune levels (Fig. 2A). A phage display library was constructed with a diversity of 1.0 × 10^8^ CFU. Colony PCR analysis of 24 randomly selected clones showed a 100% insertion rate of the VHH gene (~ 400 bp), indicating high library quality (Fig. 2B).

### Screening and identification of PEDV specific nanobodies

**Fig. 3:**
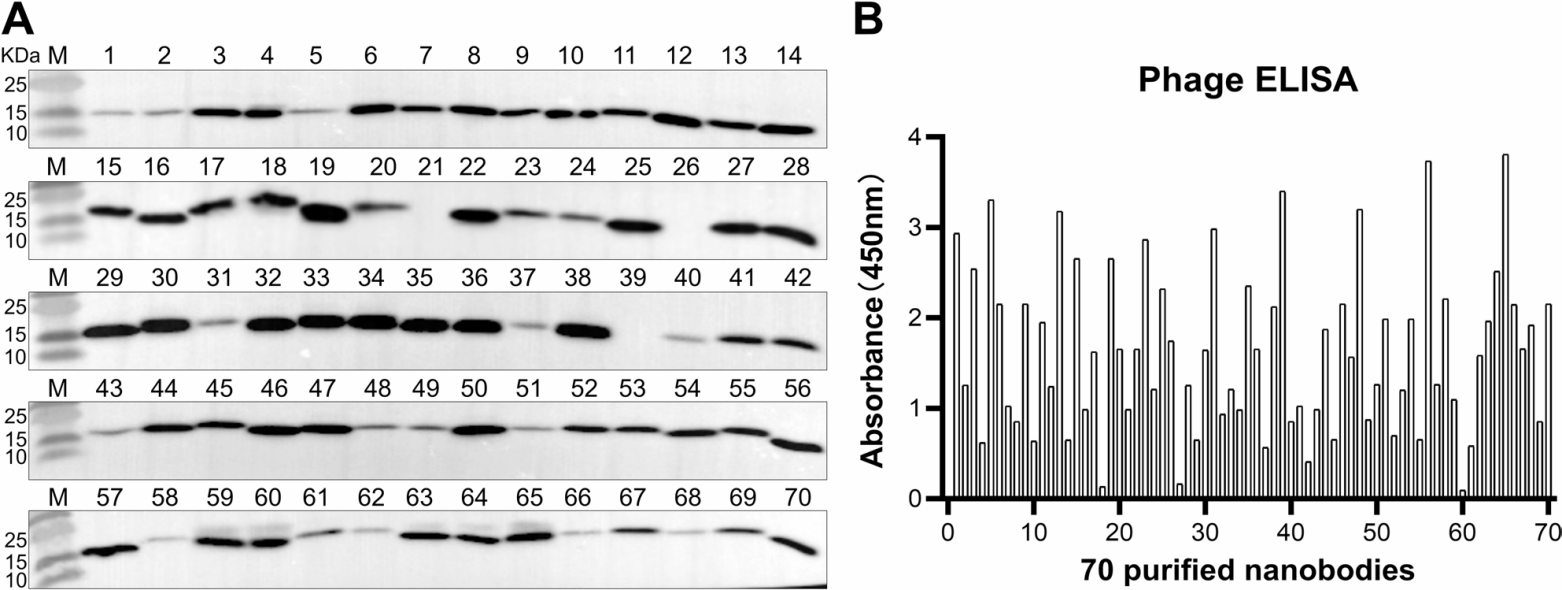
Identification and initial characterization of PEDV S-specific nanobodies. **A**: SDS-PAGE analysis of representative purified nanobodies expressed in *E. coli*. M: Protein molecular weight marker; 1 ~ 70: 70 purified nanobodies. **B**: Phage ELISA analysis of the binding reactivity of selected unique nanobodies to the recombinant PEDV S protein

After three rounds of biopanning, we screened 192 individual colonies by PE-ELISA, identifying 106 positive binders. Sequence analysis revealed 70 unique nanobodies based on CDR3 diversity. These unique clones were expressed and purified from *E. coli*. Western Blot confirmed that most nanobodies (67/70) were soluble and purified to > 90% homogeneity (Fig. 3A). Phage ELISA further verified that these purified nanobodies maintained strong binding activity towards the S protein (Fig. 3B).

### Binding specificity of selected nanobodies

**Fig. 4:**
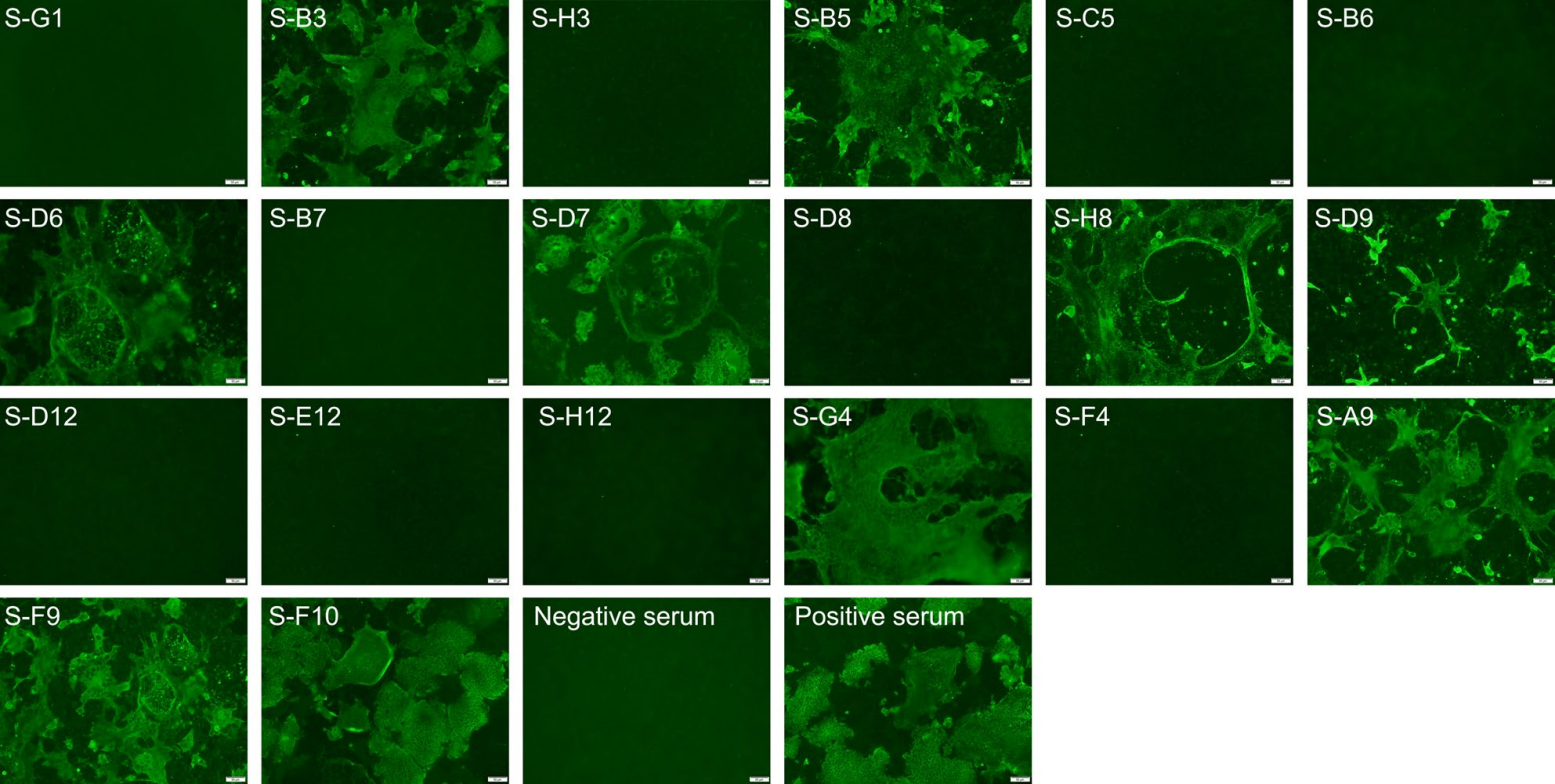
Indirect immunofluorescence assay (IFA) of PEDV-infected Vero cells stained with nanobody-Fc fusion proteins. Green fluorescence indicates specific binding of nanobodies to viral antigens in the perinuclear region. Nuclei were counterstained with DAPI. Positive control: Serum from PEDV-vaccinated pigs; negative control: serum from healthy, unvaccinated pigs

**Fig. 5:**
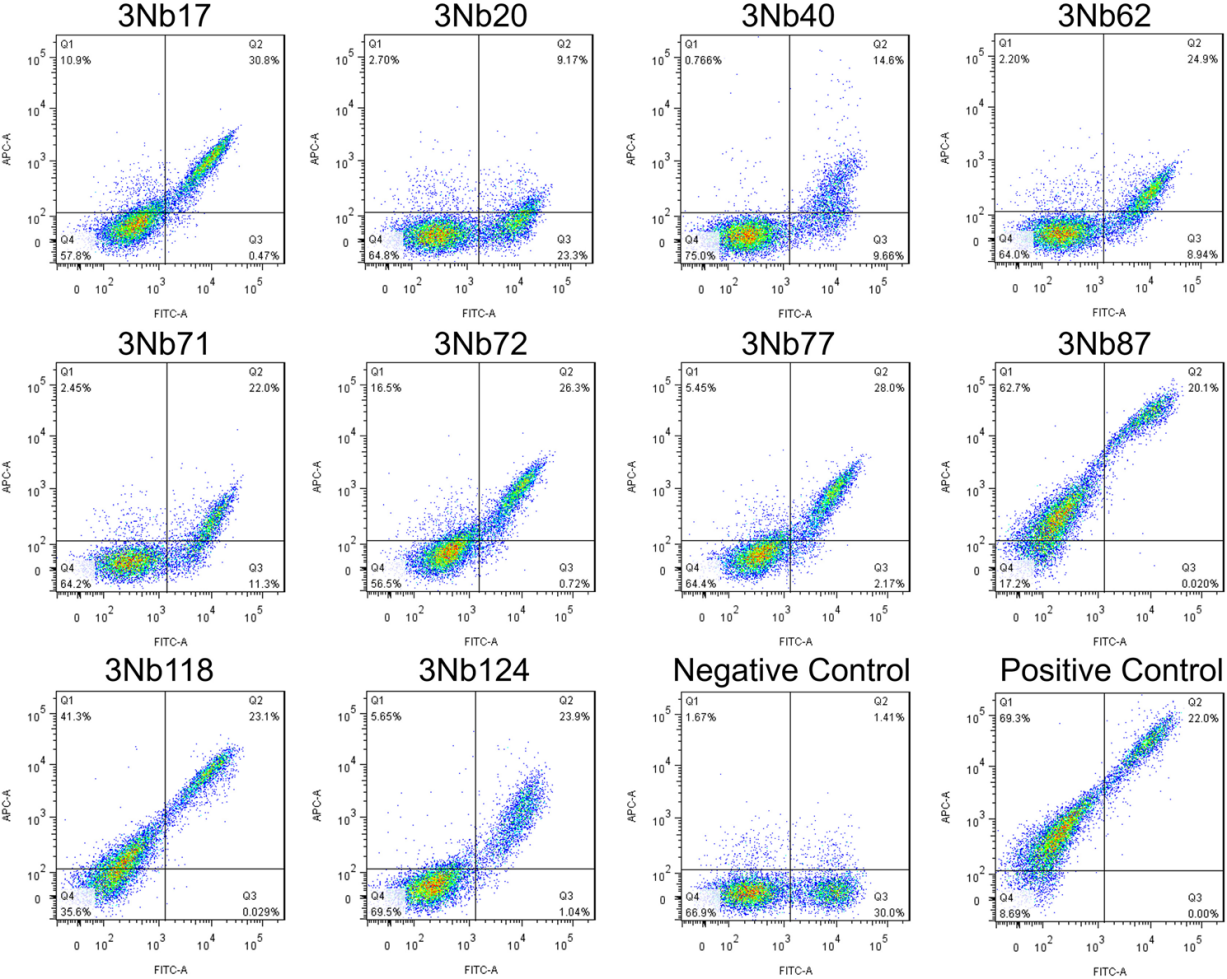
Flow cytometric analysis of the specific binding of nanobodies to cell-surface expressed PEDV S protein. The binding of nanobodies was detected using an APC-conjugated anti-mFc antibody (Y-axis), and S protein expression was verified using an anti-His FITC-conjugated antibody (X-axis). Cells in the upper right quadrant (Q2, FITC^+^/APC^+^) represent the double-positive population, indicating successful binding of nanobodies to the expressed S protein. Positive control: Serum from PEDV-vaccinated pigs; negative control: an irrelevant nanobody

18 high-affinity nanobodies were selected for further characterization. In IFA, 10 nanobodies (including 3Nb17, 3Nb20, 3Nb40, 3Nb62, 3Nb71, 3Nb72, 3Nb77, 3Nb87, 3Nb118, and 3Nb124) specifically recognized native viral antigens in PEDV-infected Vero cells, showing distinct perinuclear fluorescence (Fig. 4). Flow cytometry analysis demonstrated that 9 of these 10 nanobodies could efficiently bind to the S protein expressed on the HEK293T cell surface (Fig. 5), characterized by a high percentage of double-positive cells (FITC+/APC + in the Q2 quadrant). Notably, 3Nb20 exhibited a weakly positive binding signal. The robust binding observed for the majority of the candidates suggests they target surface-exposed epitopes critical for viral attachment.

### Neutralization potency and affinity of 3Nb17

**Fig. 6:**
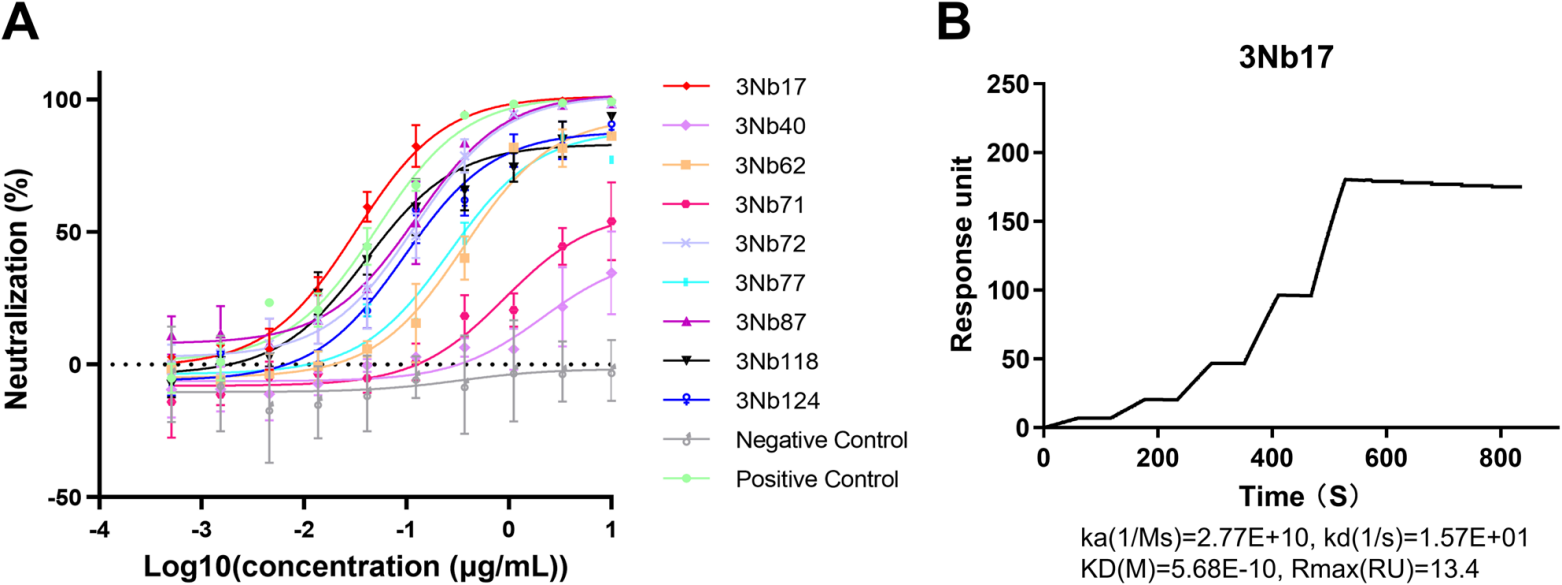
Neutralization potency and binding kinetics of the lead candidate 3Nb17. **A**: In vitro microneutralization assay of 3Nb17 against PEDV CH-HB2-2018 strain in Vero cells. The graph shows the percentage of viral inhibition at varying nanobody concentrations. The half-maximal inhibitory concentration (IC_50_) was calculated by non-linear regression. **B**: Surface Plasmon Resonance (SPR) sensorgram characterizing the affinity of 3Nb17. The purified nanobody was injected over immobilized PEDV S protein at various concentrations. The black curves represent experimental data, and the red curves represent the global fit to a 1:1 Langmuir binding model. The calculated equilibrium dissociation constant (*K*_D_) is shown

The 9 nanobodies that exhibited strong binding in flow cytometry analysis were further tested for virus neutralization. Among them, nanobody 3Nb17 exhibited the most potent antiviral activity, effectively inhibiting PEDV-induced CPE in a dose-dependent manner (Fig. 6A). The calculated IC_50_ for 3Nb17 was 0.0318 µg/mL. To elucidate the basis of this potency, we measured its binding kinetics via SPR. 3Nb17 displayed a fast association rate (*K*_on_) and a slow dissociation rate (*K*_off_), resulting in a high affinity constant (*K*_D_) of 0.568 nM (Fig. 6B). These data identify 3Nb17 as a promising lead candidate for therapeutic development.

## Discussion

Porcine epidemic diarrhea virus (PEDV) remains a recalcitrant pathogen in the global swine industry. Despite the availability of inactivated and attenuated vaccines, outbreaks continue to occur periodically [23]. A primary challenge lies in the high genetic plasticity of the spike (S) protein, which facilitates viral escape from vaccine-induced immunity [24]. Consequently, there is a pressing need for broad-spectrum, high-affinity therapeutic agents that can complement existing prophylactic measures.

In this study, we targeted the S protein rather than the nucleocapsid (N) protein for nanobody selection. While N protein is highly immunogenic, antibodies against it are generally non-neutralizing [25]. More critically, anti-N antibodies have been implicated in the antibody-dependent enhancement (ADE) of infection in other coronaviruses. It is noteworthy that even S-protein-based subunit vaccines can induce ADE in piglets if they fail to elicit high titers of neutralizing antibodies, as reported by Yu et al. [9]. This highlights the importance of selecting high-affinity binders that target specific neutralizing epitopes on the S1 subunit to block viral entry effectively while minimizing safety risks [26].

Our study successfully isolated a panel of neutralizing nanobodies, with 3Nb17 emerging as the lead candidate. 3Nb17 exhibited a picomolar binding affinity (*K*_D_ ≈ 0.568 nM) and a potent IC_50_ of 0.0318 µg/mL against the prevalent CH-HB2-2018 strain. These parameters are comparable to or superior to several previously reported monoclonal antibodies. The superior performance of 3Nb17 likely stems from the unique structural features of nanobodies. Their convex paratope and long CDR3 loop allow them to penetrate the heavy glycan shield of the PEDV S protein and access conserved receptor-binding pockets that are often sterically inaccessible to conventional IgG molecules [15, 27].

However, this study has limitations. First, the screening was conducted against a single PEDV genotype (GIIb). Given the co-circulation of multiple strains (e.g., S-INDEL variants), future work must evaluate the cross-neutralizing breadth of 3Nb17 [2]. Second, while in vitro results are promising, the in vivo protective efficacy and pharmacokinetics of 3Nb17 need to be validated in neonatal piglet challenge models. Finally, although we successfully expressed soluble nanobodies, their half-life in serum is naturally short due to their small size. Developing half-life extended formats, such as fusion with albumin-binding domains or Fc regions, will be essential for their clinical application [28].

## Conclusion

In summary, we have established a robust pipeline for the generation of antiviral nanobodies and identified 3Nb17 as a potent neutralizer of PEDV. With its exceptional affinity, stability, and high production yield in prokaryotic systems, 3Nb17 represents a promising candidate for the development of next-generation diagnostic kits and therapeutic interventions. Future structural studies using cryo-electron microscopy will further elucidate its mechanism of action, paving the way for the rational design of multivalent nanobody cocktails to combat viral evolution.

## Supplementary Information

Supplementary Material 1. The uncropped images of electrophoretic gel, SDS-PAGE, and Western blot have been provided as additional supporting files.

## Data Availability

The nucleotide sequences of the nanobodies (3Nb17, 3Nb20, 3Nb40, 3Nb62, 3Nb71, 3Nb72, 3Nb77, 3Nb87, 3Nb118, and 3Nb124) generated during the current study are available in the GenBank repository under accession numbers PZ261600 to PZ261609 (https://www.ncbi.nlm.nih.gov/nuccore/PZ261600:PZ261609).

## References

[1] Zhang Y, Chen Y, Zhou J, Wang X, Ma L, Li J, Yang L, Yuan H, Pang D, Ouyang H. Porcine epidemic diarrhea virus: an updated overview of virus epidemiology, virulence variation patterns and virus-host interactions. Viruses. 2022;14(11):2434. 10.3390/v14112434. 10.3390/v14112434 PMC9695474 36366532

[2] Hu G, Kang Q, Luo Z, Geng R, Zhao Z, Peng O, Zou C, Feng S, Cao Y, Shen H, et al. Characterization and pathogenic evaluation of a novel S-INDEL PEDV CH/JSHA2024 isolated in China. Anim Dis. 2025;5:19. 10.1186/s44149-025-00174-x.

[3] Huang B, Gu G, Zhang Y, Chen Z, Tian K. Molecular and structural evolution of porcine epidemic diarrhea virus. Animals. 2022;12(23):3388. 10.3390/ani12233388. 10.3390/ani12233388 PMC9736354 36496909

[4] Luo H, Liang Z, Lin J, Wang Y, Liu Y, Mei K, Zhao M, Huang S. Research progress of porcine epidemic diarrhea virus S protein. Front Microbiol. 2024;15:1396894. 10.3389/fmicb.2024.1396894. 10.3389/fmicb.2024.1396894 PMC11169810 38873162

[5] Zhou C, Liu Y, Wei Q, Chen Y, Yang S, Cheng A, Zhang G. HSPA5 Promotes attachment and internalization of porcine epidemic diarrhea virus through interaction with the spike protein and the endo-/lysosomal pathway. J Virol. 2023;97(6):e00549–23. 10.1128/jvi.00549-23. 37222617 10.1128/jvi.00549-23 PMC10308931

[6] Zaichuk TA, Nechipurenko YD, Adzhubey AA, Onikienko SB, Chereshnev VA, Zainutdinov SS, Kochneva GV, Netesov SV, Matveeva OV. The challenges of vaccine development against betacoronaviruses: antibody dependent enhancement and Sendai virus as a possible vaccine vector. Mol Biol. 2020;54(6):812–26. 10.1134/s0026893320060151. 32921819 10.1134/S0026893320060151 PMC7473411

[7] Nakayama EE, Shioda T. Detrimental effects of anti-nucleocapsid antibodies in SARS-CoV-2 infection, reinfection, and the post-acute sequelae of COVID-19. Pathogens. 2024;13(12):1109. 10.3390/pathogens13121109. 39770368 10.3390/pathogens13121109 PMC11728538

[8] Li C, Li W, Lucio de Esesarte E, Guo H, van den Elzen P, Aarts E, van den Born E, Rottier PJM, Bosch B-J. Cell attachment domains of the porcine epidemic diarrhea virus spike protein are key targets of neutralizing antibodies. J Virol. 2017;91(12):e00273–17. 10.1128/jvi.00273-17. 28381581 10.1128/JVI.00273-17 PMC5446644

[9] Yu J, Sreenivasan C, Uprety T, Gao R, Huang C, Lee EJ, Lawson S, Nelson J, Christopher-Hennings J, Kaushik RS, et al. Piglet immunization with a spike subunit vaccine enhances disease by porcine epidemic diarrhea virus. NPJ Vaccines. 2021;6(1):22. 10.1038/s41541-021-00283-x. 10.1038/s41541-021-00283-x PMC7851141 33526776

[10] Qian Q, Zhao S, Yang L, Xing G, Chen Y, Liang C, Wang H, Li R, Qiao S, Wang A, et al. Palmitoylation enhances the stability of porcine epidemic diarrhea virus spike protein by antagonizing its degradation via chaperone-mediated autophagy to facilitate viral proliferation. J Virol. 2025;99(6):e00347–25. 10.1128/jvi.00347-25. 40401979 10.1128/jvi.00347-25 PMC12172468

[11] Zhu H, Lou J, Yang Z, Bai J, Jiang P, Wang X, Liu X. STT3B promotes porcine epidemic diarrhea virus replication by regulating N-glycosylation of PEDV S protein. J Virol. 2025;99(3):e00018–25. 10.1128/jvi.00018-25. 39945486 10.1128/jvi.00018-25 PMC11915848

[12] Su Q, Shi W, Huang X, Yin S, Yang X, Lu X. Recent advances of nanobody applications in diagnosis and detection. MedComm Biomater Appl. 2023;2(3):54. 10.1002/mba2.54.

[13] Joly S, Augusto G, Mdzomba B, Meli I, Vogel M, Chan A, Pernet V. Nogo-A neutralization in the central nervous system with a blood-brain barrier-penetrating antibody. J Control Release. 2024;366:52–64. 10.1016/j.jconrel.2023.12.041. 38154541 10.1016/j.jconrel.2023.12.041

[14] Hao Z, Dong X, Zhang Z, Qin Z. A nanobody of PEDV S1 protein: Screening and expression in Escherichia coli. Biomolecules. 2024;14(9):1116. 10.3390/biom14091116. 39334881 10.3390/biom14091116 PMC11430113

[15] Tang H, Gao Y, Han J. Application progress of the single domain antibody in medicine. Int J Mol Sci. 2023;24(4):4176. 10.3390/ijms24044176. 36835588 10.3390/ijms24044176 PMC9967291

[16] Wang J, Shi B, Chen H, Yu M, Wang P, Qian Z, Hu K, Wang J. Engineered multivalent nanobodies efficiently neutralize SARS-CoV-2 Omicron subvariants BA.1, BA.4/5, XBB.1 BQ.1.1. Vaccines. 2024;12(4):417. 10.3390/vaccines12040417. 38675799 10.3390/vaccines12040417 PMC11054741

[17] Zhao Y, Fan B, Song X, Gao J, Guo R, Yi C, He Z, Hu H, Jiang J, Zhao L, et al. PEDV-spike-protein-expressing mRNA vaccine protects piglets against PEDV challenge. mBio. 2024;15(2):e0295823. 10.1128/mbio.02958-23. 38231557 10.1128/mbio.02958-23 PMC10865985

[18] Mark JM, David IB, Patricia W, Richard R, Evan A, Nadine R, Michelle D, Jack TS, Srilatha E, Paul S, et al. Serological Responses to an Avian Influenza A/H7N9 Vaccine Mixed at the Point-of-Use With MF59 Adjuvant. JAMA. 2014;312(14):1409–19. 10.1001/jama.2014.12854. 25291577 10.1001/jama.2014.12854

[19] Vincke C, Gutiérrez C, Wernery U, Devoogdt N, Hassanzadeh-Ghassabeh G, Muyldermans S. Generation of single domain antibody fragments derived from camelids and generation of manifold constructs. Methods Mol Biol. 2012;907:145–76. 10.1007/978-1-61779-974-7_8. 22907350 10.1007/978-1-61779-974-7_8

[20] Burleson FG, Chambers TM, Wiedbrauk DL. Virology: a laboratory manual. London: Academic Press; 1992. pp. 58–61.

[21] Reed LJ, Muench H. A simple method of estimating fifty per cent endpoints. Am J Epidemiol. 1938;27:493–7.

[22] Hearty S, Leonard P, O’Kennedy R. Measuring antibody-antigen binding kinetics using surface plasmon resonance. Methods Mol Biol. 2012;901:411–42. 10.1007/978-1-61779-974-7_24. 10.1007/978-1-61779-974-7_24 22907366

[23] Yu R, Dong S, Chen B, Si F, Li C. Developing next-generation live attenuated vaccines for porcine epidemic diarrhea using reverse genetic techniques. Vaccines. 2024;12(5):557. 10.3390/vaccines12050557. 38793808 10.3390/vaccines12050557 PMC11125984

[24] Lin CM, Saif LJ, Marthaler D, Wang Q. Evolution, antigenicity and pathogenicity of global porcine epidemic diarrhea virus strains. Virus Res. 2016;226:20–39. 10.1016/j.virusres.2016.05.023. 10.1016/j.virusres.2016.05.023 PMC7111424 27288724

[25] Jung K, Saif LJ, Wang Q. Porcine epidemic diarrhea virus (PEDV): An update on etiology, transmission, pathogenesis, and control strategies. Virus Res. 2020;286:198045. 10.1016/j.virusres.2020.198045. 32502552 10.1016/j.virusres.2020.198045 PMC7266596

[26] Lee WS, Wheatley AK, Kent SJ, DeKosky BJ. Antibody-dependent enhancement and SARS-CoV-2 vaccines and therapies. Nat Microbiol. 2020;5(10):1185–91. 10.1038/s41564-020-00789-5. 10.1038/s41564-020-00789-5 PMC12103240 32908214

[27] Weiss RA, Verrips CT. Nanobodies that neutralize HIV. Vaccines. 2019;7(3):77. 10.3390/vaccines7030077. 31370301 10.3390/vaccines7030077 PMC6789485

[28] Abdolvahab MH, Karimi P, Mohajeri N, Abedini M, Zare H. Targeted drug delivery using nanobodies to deliver effective molecules to breast cancer cells: the most attractive application of nanobodies. Cancer Cell Int. 2024;24(1):67. 10.1186/s12935-024-03259-8. 38341580 10.1186/s12935-024-03259-8 PMC10858526

