# Supplementary material for: Neutralizing nanobodies against porcine epidemic diarrhea virus: discovery and characterization
Source: BMC Vet Res. 2026 Jul 23;22:438. doi: 10.1186/s12917-026-05628-z (PMC13393878; doi:10.1186/s12917-026-05628-z)

Supplementary figures

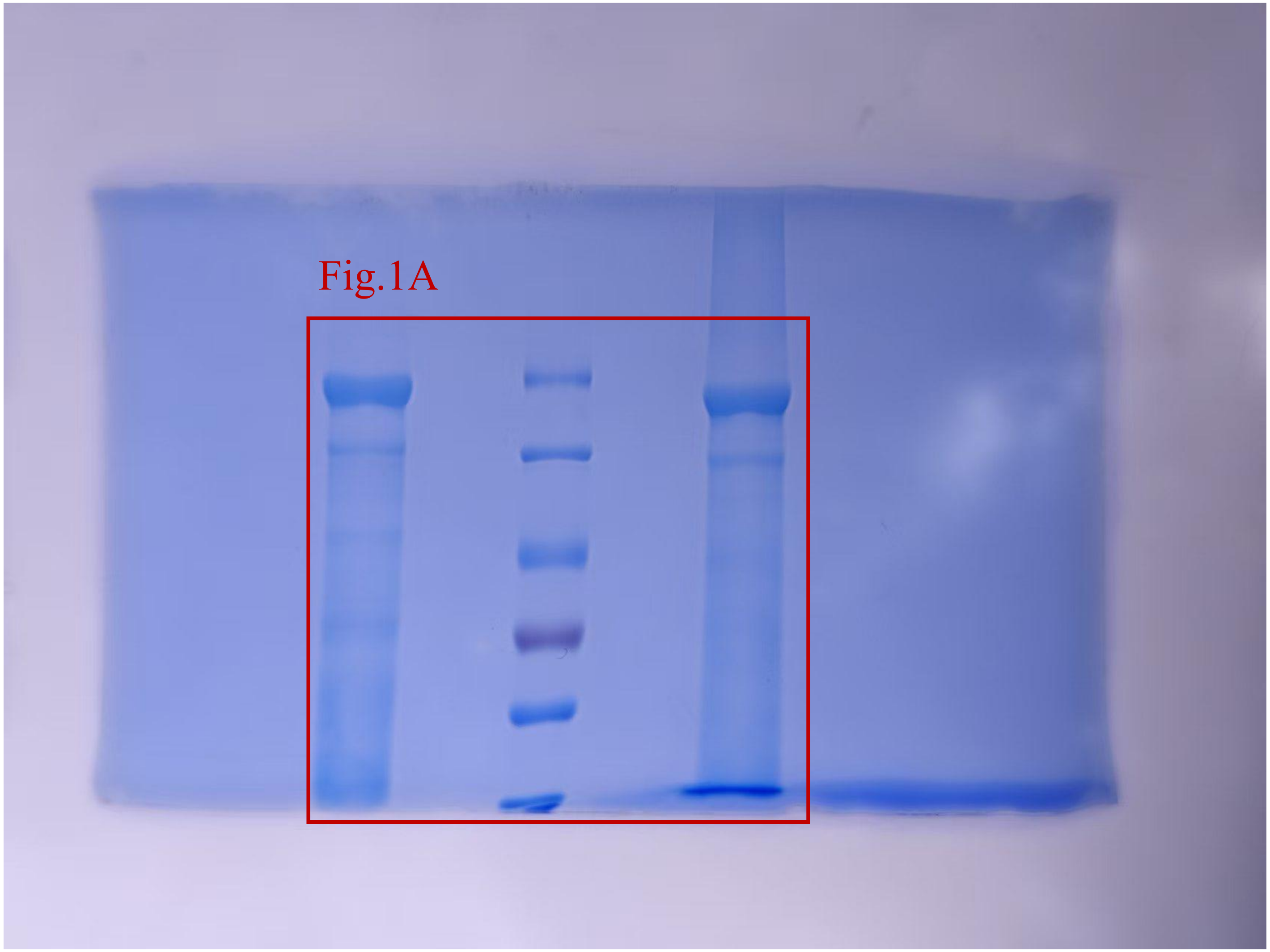

Supplementary figures

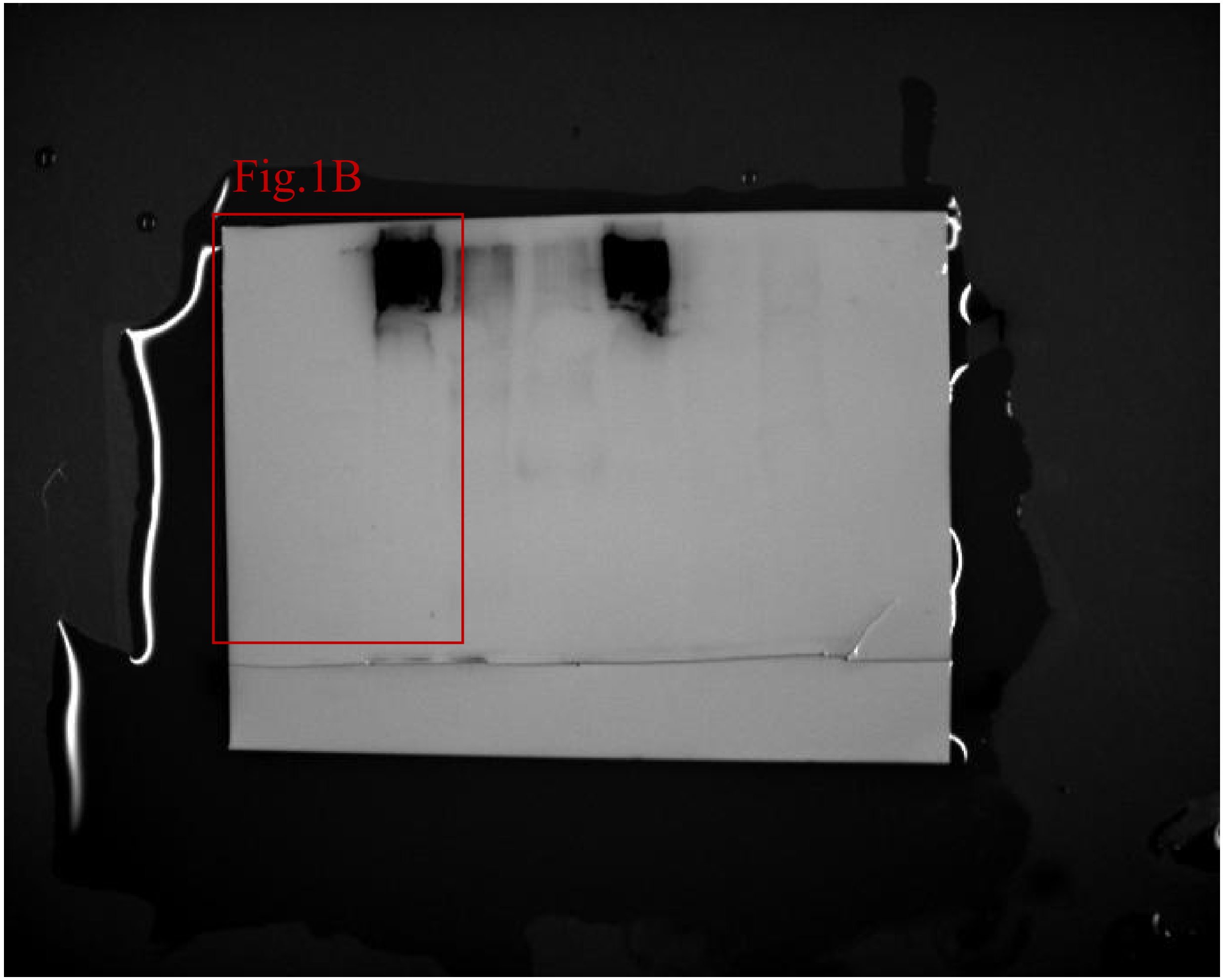

Supplementary figures

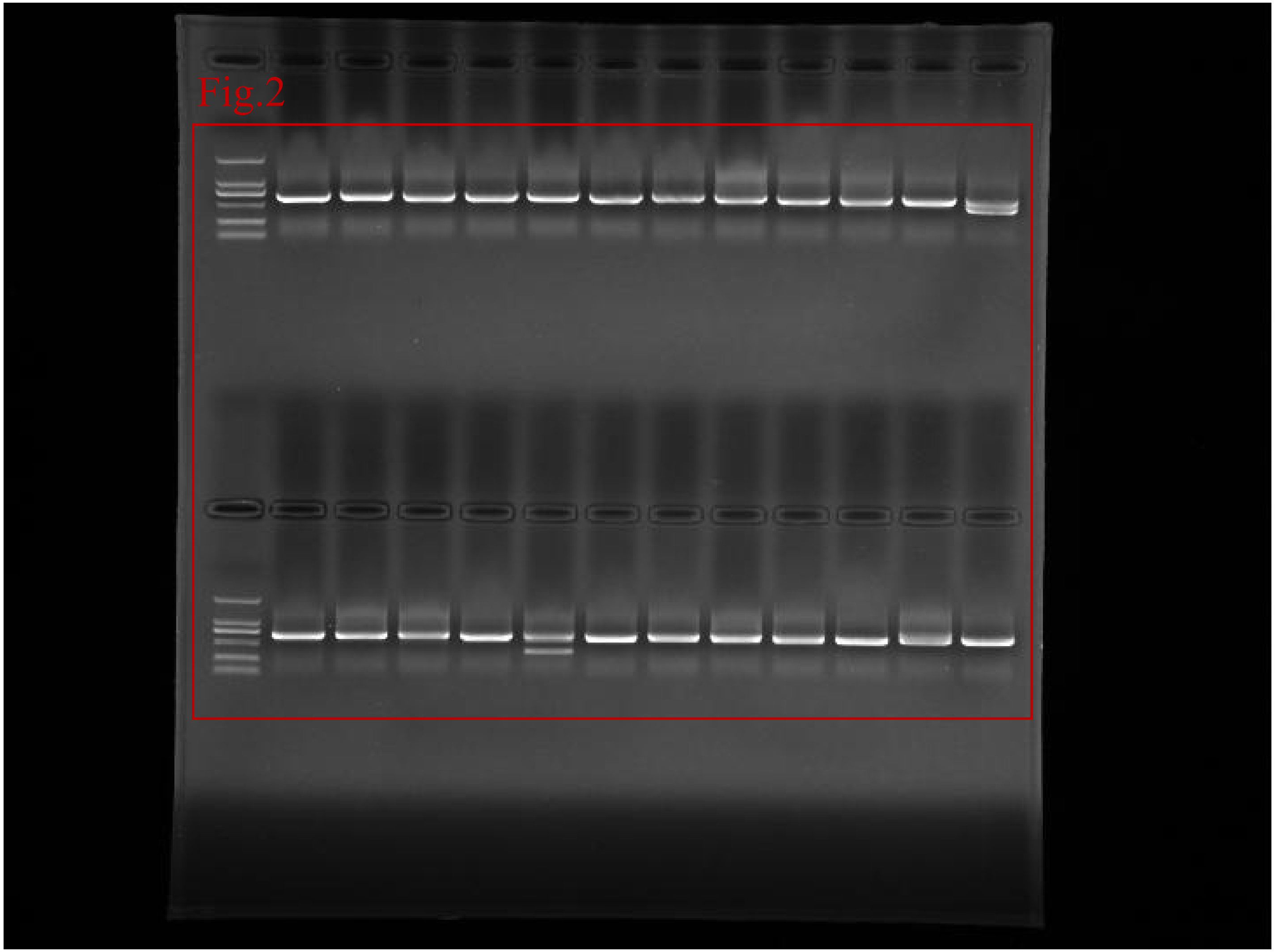

Supplementary figures

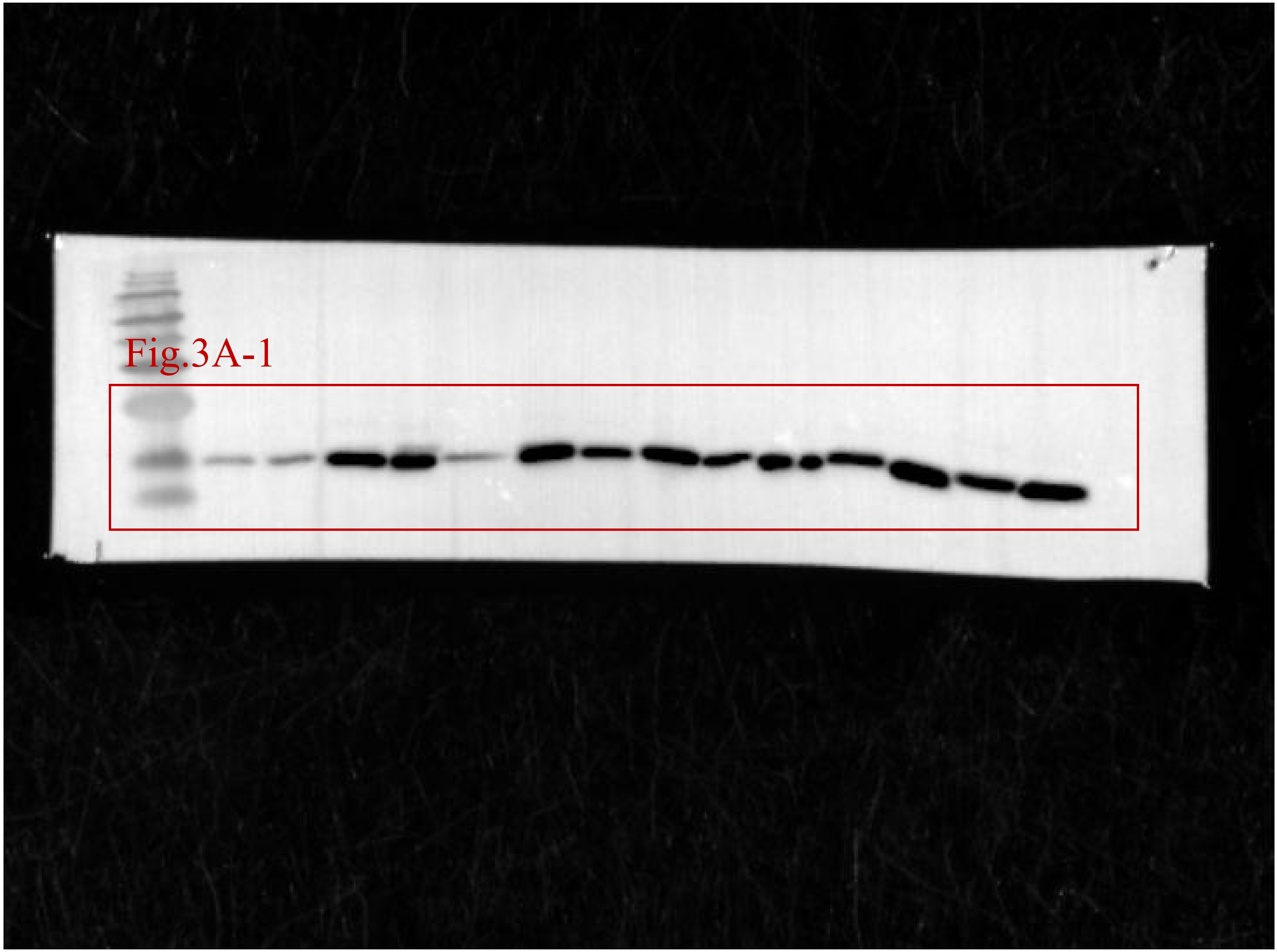

Supplementary figures

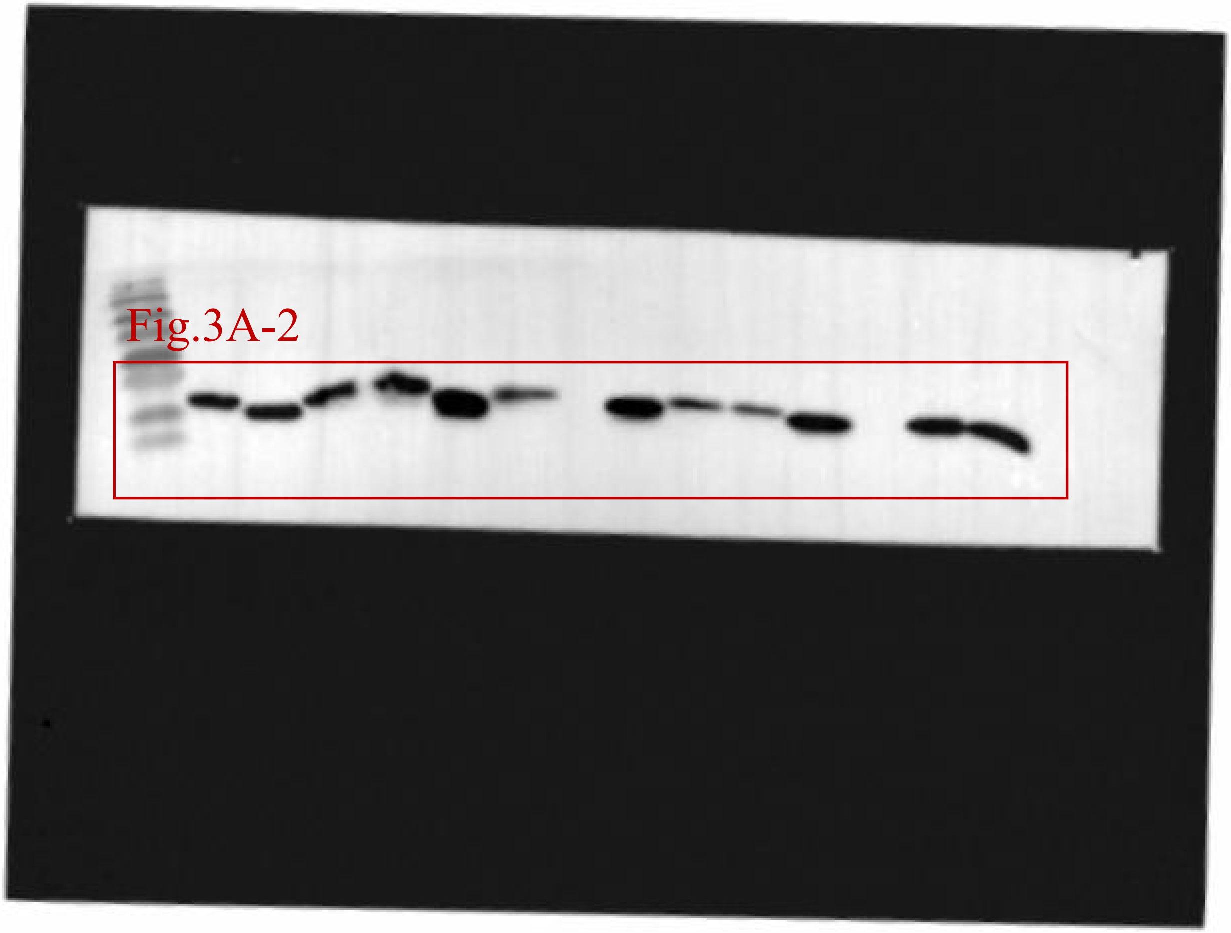

Supplementary figures

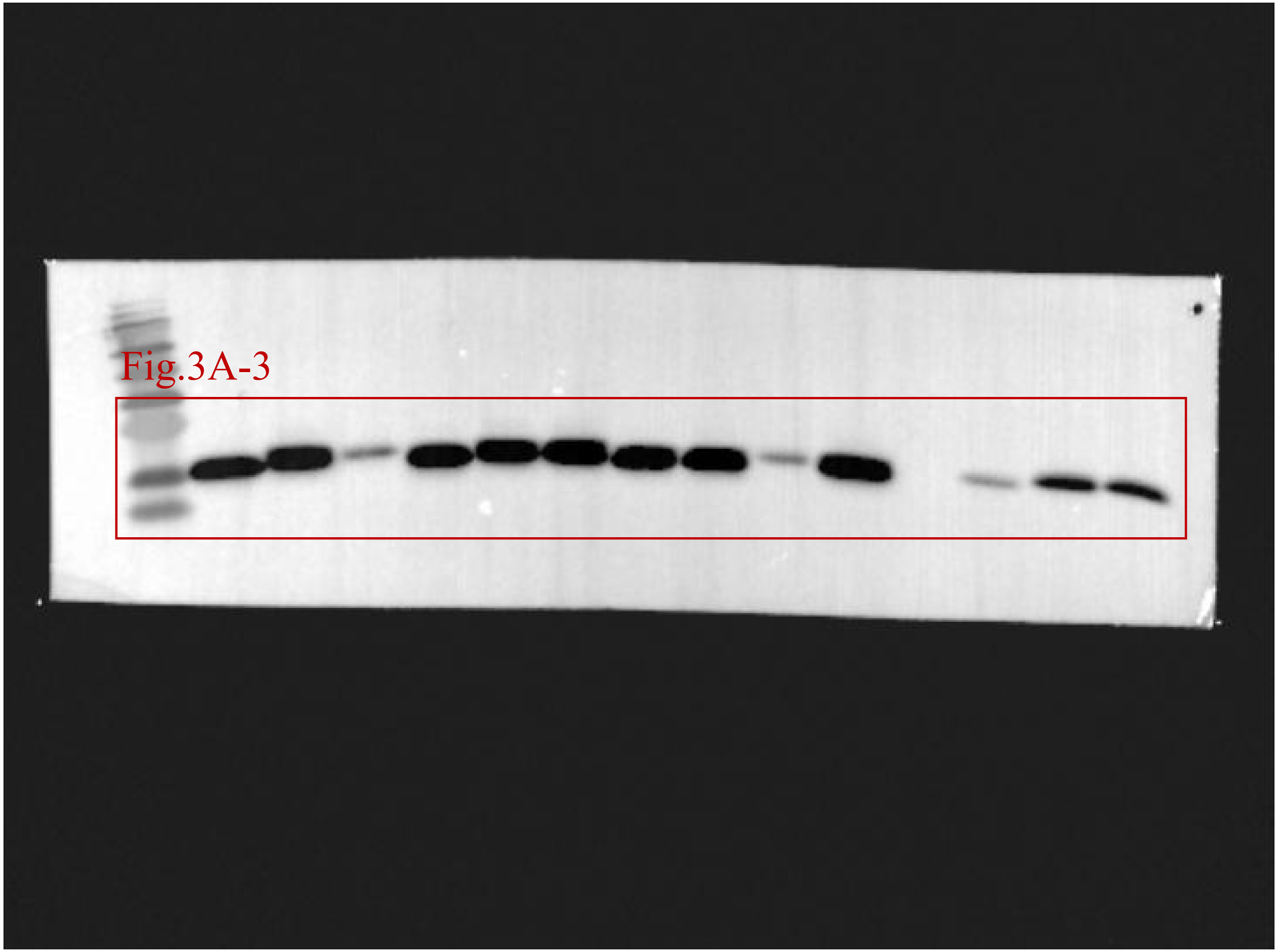

Supplementary figures

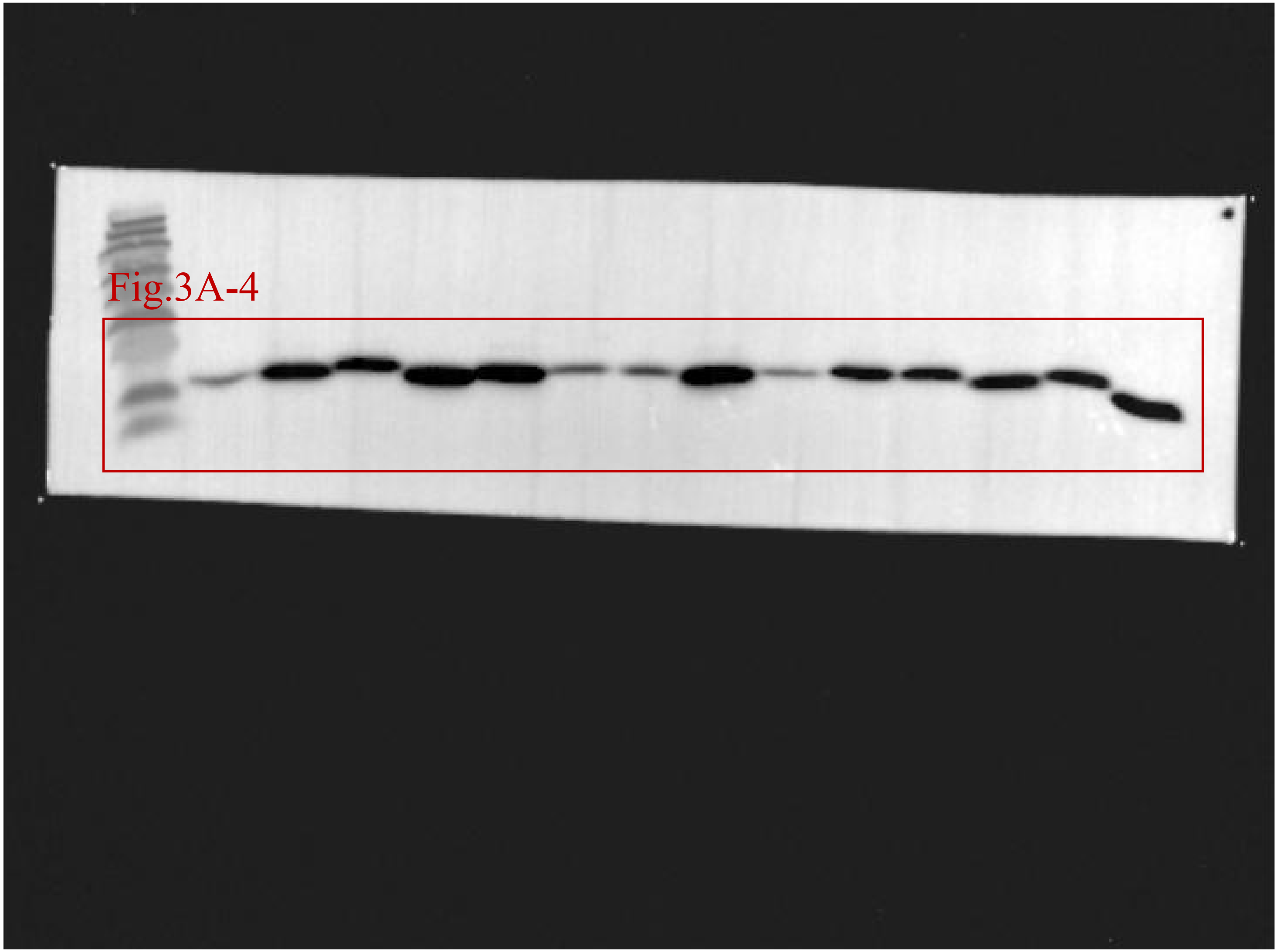

Supplementary figures

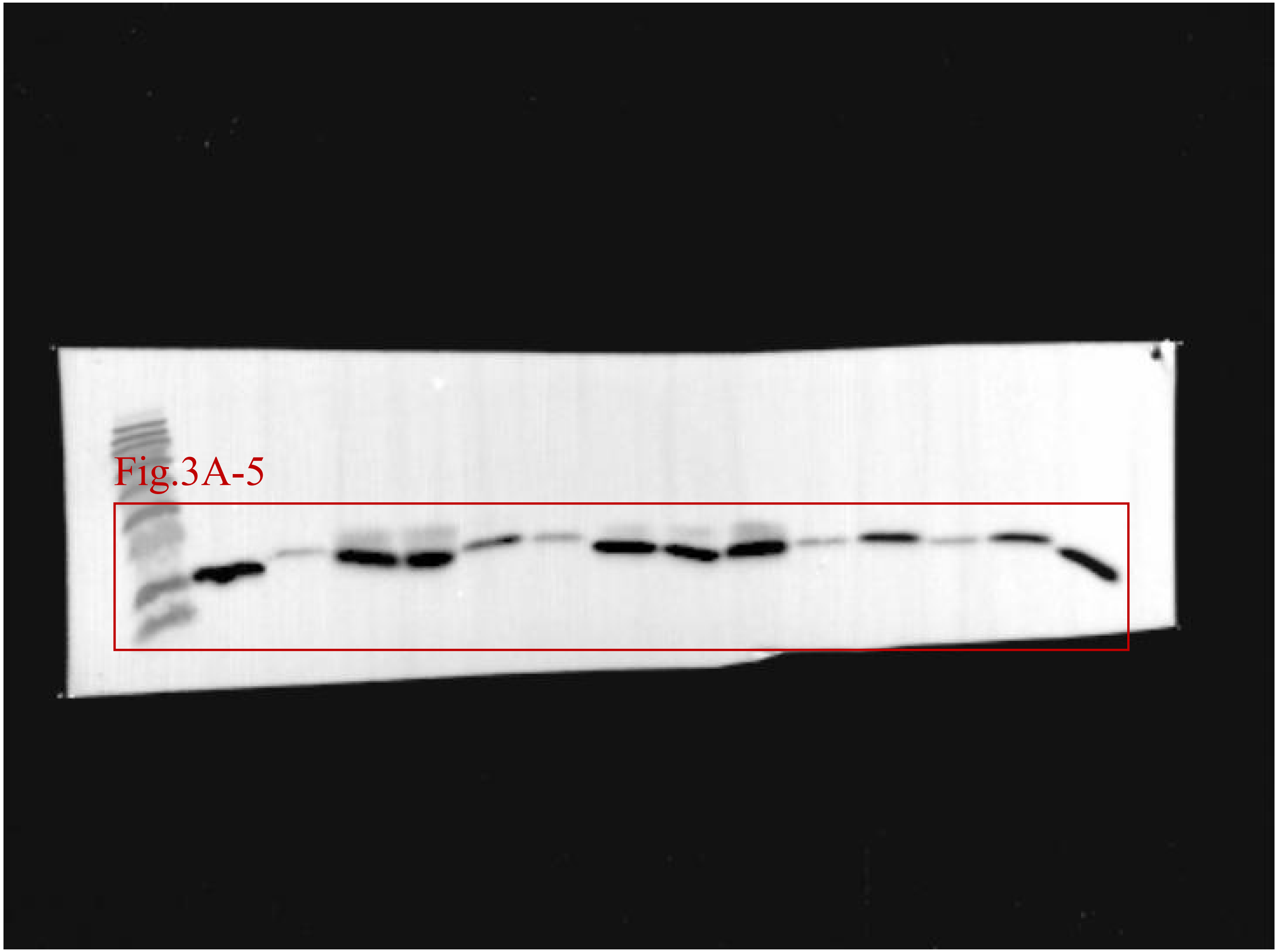

Supplement: Supplementary file 1 — Supplementary Material 1. The uncropped images of electrophoretic gel, SDS-PAGE, and Western blot have been provided as additional supporting files. [file 12917_2026_5628_MOESM1_ESM.pdf]
